# Using ELISPOT to Expose False Positive Skin Test Conversion in Tuberculosis Contacts

**DOI:** 10.1371/journal.pone.0000183

**Published:** 2007-01-31

**Authors:** Philip C. Hill, David J. Jeffries, Roger H. Brookes, Annette Fox, Dolly Jackson-Sillah, Moses D. Lugos, Simon A. Donkor, Bouke C. de Jong, Tumani Corrah, Richard A. Adegbola, Keith P. McAdam

**Affiliations:** Bacterial Diseases Programme, Medical Research Council Unit, Banjul, The Gambia; Columbia University, United States of America

## Abstract

**Background:**

Repeat tuberculin skin tests may be false positive due to boosting of waned immunity to past mycobacterial exposure. We evaluated whether an ELISPOT test could identify tuberculosis (TB) contacts with boosting of immunity to non-tuberculous mycobacterial exposure.

**Methodology/Principal Findings:**

We conducted tuberculin and ELISPOT tests in 1665 TB contacts: 799 were tuberculin test negative and were offered a repeat test after three months. Those with tuberculin test conversion had an ELISPOT, chest X-ray and sputum analysis if appropriate. We compared converters with non-converters, assessed the probability of each of four combinations of ELISPOT results over the two time points and estimated boosting with adjustment for ELISPOT sensitivity and specificity. 704 (72%) contacts had a repeat tuberculin test; 176 (25%) had test conversion, which increased with exposure to a case (p = 0.002), increasing age (p = 0.0006) and BCG scar (p = 0.06). 114 tuberculin test converters had ELISPOT results: 16(14%) were recruitment positive/follow-up positive, 9 (8%) positive/negative, 34 (30%) negative/positive, and 55 (48%) were negative/negative. There was a significant non-linear effect of age for ELISPOT results in skin test converters (p = 0.038). Estimates of boosting ranged from 32%–41% of skin test converters with increasing age. Three converters were diagnosed with TB, two had ELISPOT results: both were positive, including one at recruitment.

**Conclusions/Significance:**

We estimate that approximately one third of tuberculin skin test conversion in Gambian TB case contacts is due to boosting of immunity to non-tuberculous mycobacterial exposure. Further longitudinal studies are required to confirm whether ELISPOT can reliably identify case contacts with tuberculin test conversion that would benefit most from prophylactic treatment.

## Introduction

The period of highest risk for developing tuberculosis (TB) is in the first year after exposure to *Mycobacterium tuberculosis*.[1] Individuals most likely to be in this group are those who have a negative test for *M. tuberculosis* infection, exposure to a TB case and subsequent test conversion. However, conversion of the traditional tuberculin skin test can be confounded by the ‘booster’ phenomenon, whereby an initial tuberculin injection causes recall of waned cell-mediated immunity to previous, largely non-tuberculous, mycobacterial exposure. Tuberculin probably does not cause a truly ‘mycobacterially naïve’ person to become positive on a subsequent test.[2]

Recently, the British National Institute for Health and Clinical Excellence (NICE) published recommendations suggesting that the traditional tuberculin test and an interferon-gamma test be used in a two-step manner – the tuberculin test as a screening tool and an interferon-gamma test as confirmation.[3] Such a strategy has not been assessed in practice. It is our view that such a two-step approach may be most appropriate when trying to distinguish true tuberculin skin test conversion from that due to the booster phenomenon, at least in relation to previous non-tuberculous exposure. One would expect such a phenomenon to occur most frequently where there is a high rate of BCG vaccination at birth and intense exposure to environmental mycobacteria, as these are particularly associated with early skin test reversion [4], [5]. The Gambia is such a setting.

T cell based interferon-gamma assays that incorporate stimulatory antigens that are not found in BCG or many environmental mycobacteria, have been shown to have promise in the diagnosis of *M. tuberculosis* infection after recent exposure.[6], [7], [8] Here we assess whether ELISPOT has utility in distinguishing tuberculin skin test converters with true *M. tuberculosis* infection from those with boosting of immunity to prior, non-tuberculous, mycobacterial exposure.

## Methods

### Participants

Consecutive recruitment of sputum smear and culture positive TB cases and their household contacts in The Gambia, plus the selection process for ELISPOT testing, have been described previously.[7] Household contacts of TB cases were eligible for inclusion in this study if they were at least 6 months old and had a recruitment ELISPOT result but were not diagnosed with TB disease. The study was approved by the combined Gambia Government/MRC ethics committee.

Contacts were interviewed, examined, and a blood sample taken for ELISPOT and HIV test. Immediately after the blood sample was taken they underwent a tuberculin skin test (2 TU, PPD RT23, Statens Serum Institut, Copenhagen, Denmark). Those who were tuberculin test negative at recruitment (<10 mm of induration) were asked to have a repeat tuberculin test at 3 months. Tuberculin test conversion was defined as a positive test (> = 10 mm induration) plus an increase in induration of at least 6 mm. All tuberculin test converters were asked to have a chest x-ray and a clinical examination. Those able to produce sputum underwent sputum analysis. Those diagnosed with TB disease were referred to the National Programme for free treatment. HIV positive individuals were referred to the MRC HIV clinic, where they are followed and considered for free anti-retroviral treatment. There is no current practice of preventive treatment in The Gambia.

All tuberculin test converters were asked to have a repeat ELISPOT test. To assess the effect of the tuberculin skin test on ELISPOT conversion we recruited 32 adult male volunteers from the general community in The Gambia for tuberculin test and ELISPOT test. Those that had a negative tuberculin test and negative ELISPOT test were asked to have a repeat ELISPOT test after 1 week and 1 month.

### Laboratory procedures

Sputum smears were prepared, stained and cultured,[9] plus HIV tests performed[10] as previously described. We performed ELISPOT assays in duplicate.[11] Synthetic, sequential peptides spanning the length of ESAT-6 and CFP-10 (ABC, Imperial College, London, UK) were used. Each peptide was 15 amino acids long and overlapped its adjacent peptide by 10 residues. The ESAT-6 CFP-10 peptide pools were used at a final concentration of 2.5 µg/ml for each peptide. The positive control was Phytohaemaglutinin (PHA; Sigma-Aldrich, UK). The *ex-vivo* ELISPOT assays were enumerated using an ELISPOT reader (AID-GmbH, Strasburg, Germany). Positive test wells were pre-defined as containing at least eight spot forming units (SFU) more than negative control wells.[12] For a positive result it was necessary for at least one of the pools of overlapping peptides to be positive. PHA wells were set to at least 150 SFU/well/2×10^5^ above negative control wells. Negative control wells were required to have less than 20 SFU/well/2×10^5^. ELISPOT conversion and reversion was defined as a positive test or negative test respectively, plus a change in the combined ESAT-6 and CFP-10 count (above the negative control) of at least 6 SFU/well/2×10^5^ (30 SFU/million cells). Laboratory staff were blinded as to the characteristics of the individuals tested.

For molecular sub typing of index and secondary case isolates, we extracted mycobacterial DNA using CTAB and chloroform, as previously described,[13] and assessed its concentration and purity by spectrophotometry. We performed Spoligotyping using membranes (Isogen Biosciences), as previously described,[14] scanned the results and analysed them with software designed in Matlab.

### Data management and analysis

The number of SFU in each well were automatically imported into an Access database using visual basic code and other data were double-entered into the same database and verified.[15] We assessed the relationship between conversion and reversion of tuberculin skin test results to possible risk factors using random effects logistic regression, taking into account household clustering. We included age and gender in the final multi-variable model a priori. For skin test converters with ELISPOT results, the 4 possible combinations of positive or negative baseline and 3-month follow-up ELISPOT results were fitted using multi-nomial logistic regression. This is an extension of logistic regression that is used where there are more than two possible outcomes. After fitting the predictor effects of sleeping proximity, age and BCG scar status, the best fit was determined and the predicted probabilities for each ELISPOT combination were calculated. We then estimated the proportion of skin test converters with boosting of prior non-tuberculous mycobacterial exposure, taking into account published estimates of the sensitivity and specificity of the ELISPOT.[12], [16] Statistical analyses were conducted using Stata software (version 9; Stata Corp, College Station, TX, USA).

## Results

**Figure 1:**
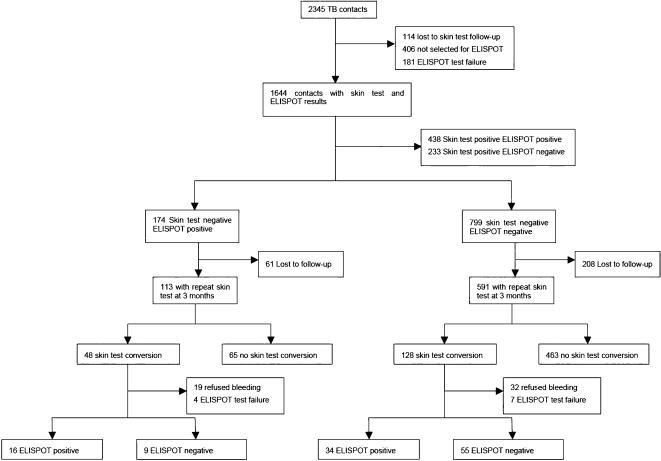
Study profile.

Between May 2002 and September 2004, 2345 contacts of 311 TB cases were recruited. Of these, 1644 (70%) were selected for an ELISPOT test in addition to a tuberculin test and had acceptable results for both (Figure 1). Of these, 799 (48.6%) were tuberculin test negative and ELISPOT negative and 174 (10.6%) were tuberculin test negative and ELISPOT positive. Table 1 shows the characteristics of these contacts and the 704 (72%) who agreed to have a repeat skin test 3 months later (table 1). Those who were tested after 3 months were slightly younger than those not tested again (mean age 17.7 years versus 22.5 years, p<0.0001) and had a slightly different ethnic mix (p = 0.038). Ninety individuals had an independent second tuberculin reading for quality control: there was agreement with respect to a positive or negative result in 88 (98%).

**Table 1:**
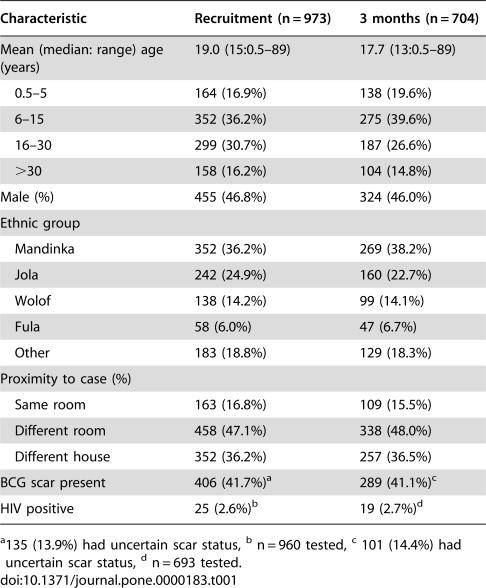
Characteristics of TB case contacts who were negative by tuberculin test and ELISPOT at recruitment and those who had a repeat skin test at 3 months.

Characteristic	Recruitment (n = 973)	3 months (n = 704)
Mean (median: range) age (years)	19.0 (15:0.5–89)	17.7 (13:0.5–89)
0.5–5	164 (16.9%)	138 (19.6%)
6–15	352 (36.2%)	275 (39.6%)
16–30	299 (30.7%)	187 (26.6%)
>30	158 (16.2%)	104 (14.8%)
Male (%)	455 (46.8%)	324 (46.0%)
Ethnic group		
Mandinka	352 (36.2%)	269 (38.2%)
Jola	242 (24.9%)	160 (22.7%)
Wolof	138 (14.2%)	99 (14.1%)
Fula	58 (6.0%)	47 (6.7%)
Other	183 (18.8%)	129 (18.3%)
Proximity to case (%)		
Same room	163 (16.8%)	109 (15.5%)
Different room	458 (47.1%)	338 (48.0%)
Different house	352 (36.2%)	257 (36.5%)
BCG scar present	406 (41.7%)^a^	289 (41.1%)^c^
HIV positive	25 (2.6%)^b^	19 (2.7%)^d^

a 135 (13.9%) had uncertain scar status, ^b^ n = 960 tested, ^c^ 101 (14.4%) had uncertain scar status, ^d^ n = 693 tested.

One hundred and seventy six (25%) initially negative contacts had tuberculin test conversion. The proportion of contacts undergoing skin test conversion increased with increasing exposure to a respective index case according to sleeping proximity (table 2, p = 0.002). It was also increased with increasing age (p = 0.0006), especially up until the age of 30 years, and in those with a visible BCG scar, although this was of borderline significance (p = 0.06). Those who were ELISPOT positive at initial screening were much more likely than those who were initially ELISPOT negative to undergo tuberculin test conversion (p = 0.0002).

**Table 2:**
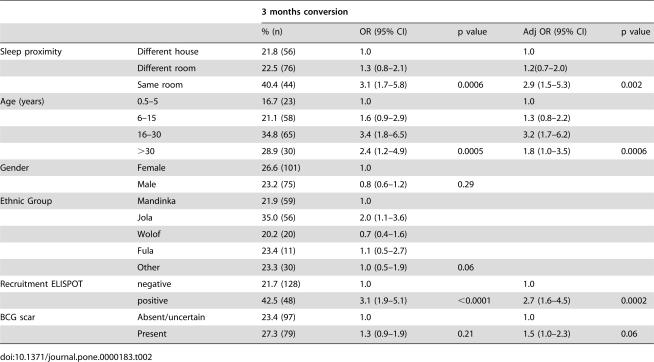
Evaluation of possible factors associated with tuberculin test conversion at 3 months in 704 TB case contacts.

		3 months conversion				
		**% (n)**	**OR (95% CI)**	**p value**	**Adj OR (95% CI)**	**p value**
Sleep proximity	Different house	21.8 (56)	1.0		1.0	
	Different room	22.5 (76)	1.3 (0.8–2.1)		1.2(0.7–2.0)	
	Same room	40.4 (44)	3.1 (1.7–5.8)	0.0006	2.9 (1.5–5.3)	0.002
Age (years)	0.5–5	16.7 (23)	1.0		1.0	
	6–15	21.1 (58)	1.6 (0.9–2.9)		1.3 (0.8–2.2)	
	16–30	34.8 (65)	3.4 (1.8–6.5)		3.2 (1.7–6.2)	
	>30	28.9 (30)	2.4 (1.2–4.9)	0.0005	1.8 (1.0–3.5)	0.0006
Gender	Female	26.6 (101)	1.0			
	Male	23.2 (75)	0.8 (0.6–1.2)	0.29		
Ethnic Group	Mandinka	21.9 (59)	1.0			
	Jola	35.0 (56)	2.0 (1.1–3.6)			
	Wolof	20.2 (20)	0.7 (0.4–1.6)			
	Fula	23.4 (11)	1.1 (0.5–2.7)			
	Other	23.3 (30)	1.0 (0.5–1.9)	0.06		
Recruitment ELISPOT	negative	21.7 (128)	1.0		1.0	
	positive	42.5 (48)	3.1 (1.9–5.1)	<0.0001	2.7 (1.6–4.5)	0.0002
BCG scar	Absent/uncertain	23.4 (97)	1.0		1.0	
	Present	27.3 (79)	1.3 (0.9–1.9)	0.21	1.5 (1.0–2.3)	0.06

One hundred and twenty-five (71%) contacts with tuberculin test conversion agreed to be re-bled for ELISPOT; 114 (91%) had an acceptable result. While 65% (n = 438 of 671; 95% confidence interval: 61.5%–68.9%) of initially tuberculin test positive contacts were ELISPOT positive, only 44% (n = 50 of 114; 95% confidence interval: 34.5%–53.5%) of tuberculin test converters were ELISPOT positive. The proportion of tuberculin test converters that were ELISPOT positive was 34.0% (18 of 53) in those with a <15 mm increase induration and 52.5% (32 of 61) of those with at least 15 mm of induration (p = 0.08). Of the 34 tuberculin test converters who were initially ELISPOT negative and had a positive repeat ELISPOT at 3 months, 32 (94%) fulfilled criteria for ELISPOT conversion. Of the 25 initially tuberculin negative, but ELISPOT positive, individuals that had tuberculin test conversion, 9 (36%) became ELISPOT negative; all 9 fulfilled criteria for ELISPOT reversion with a decrease in the combined ESAT-6/CFP-10 ELISPOT count of at least 6 spots/well.

**Figure 2:**
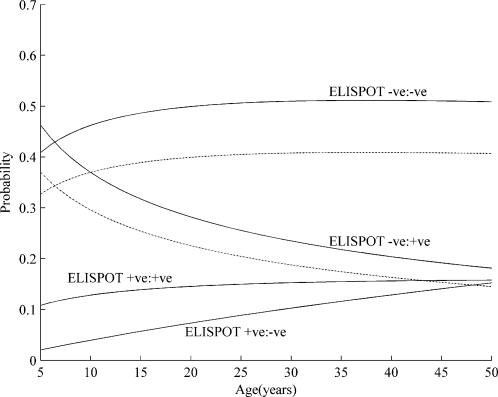
Estimates, according to age, of the probability of TB case contacts that undergo tuberculin conversion having each of the 4 possible combinations of ELISPOT results over the 2 sampling points (recruitment and after 3 months). The estimates are derived from the study data using multi-nomial logistic regression and the analysis is restricted to those aged 5–50 years (see methods). The dotted lines represent the predicted probabilities if the sensitivity (ELISPOT −ve:+ve group) and specificity (ELISPOT −ve:−ve group) of the ELISPOT are both 80% (see methods).

Using multi-nomial logistic regression we modelled the probabilities of the four possible combinations of recruitment and follow-up ELISPOT results. There was a significant non-linear effect for age (p = 0.038), but sleeping proximity (p = 0.11) and BCG scar (p-value = 0.14) were not significant. Figure 2 represents the predicted probabilities for each ELISPOT combination according to age between 5 and 50 years (outside this age range the predicted probabilities had a 95% confidence interval greater than +/−0.1). Using this figure, the proportion of tuberculin test conversion due to boosting can be estimated from the predicted probability of being ELISPOT negative at both recruitment and follow-up: allowing for a specificity adjustment (assuming 80% specificity of the ELISPOT), the proportion of skin test converters estimated to have boosting ranged from 32% to 41% with increasing age.

Of the 32 community volunteers, 14 had a negative tuberculin test and ELISPOT test. All 14 had a negative ELISPOT test again at 1 week. At one month 9 individuals agreed to be bled again and all had a negative ELISPOT test.

One hundred and forty (79.5%) skin test converters had an x-ray performed. Of these, 4 had an x-ray that was suspicious for TB and were actively investigated. Three cases were identified (table 3). They ranged from 2 to 10 years of age; all 3 were HIV negative. With respect to the one that was culture positive, the spoligotype patterns of the index and secondary case isolates were an exact match. Both of the cases with ELISPOT results at the two time points had a rise in their ELISPOT count. One had a strongly positive ELISPOT at recruitment together with 5 mm of tuberculin test induration.

**Table 3:**
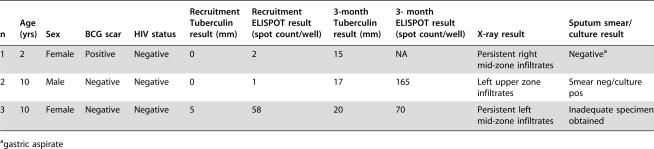
Details of tuberculin ‘converters’ who were diagnosed as secondary cases.

n	Age (yrs)	Sex	BCG scar	HIV status	Recruitment Tuberculin result (mm)	Recruitment ELISPOT result (spot count/well)	3-month Tuberculin result (mm)	3- month ELISPOT result (spot count/well)	X-ray result	Sputum smear/culture result
1	2	Female	Positive	Negative	0	2	15	NA	Persistent right mid-zone infiltrates	Negative^a^
2	10	Male	Negative	Negative	0	1	17	165	Left upper zone infiltrates	Smear neg/culture pos
3	10	Female	Negative	Negative	5	58	20	70	Persistent left mid-zone infiltrates	Inadequate specimen obtained

a gastric aspirate

## Discussion

The interval between initial exposure and tuberculin test conversion has been shown to be a maximum of 6 weeks in 99% of individuals after BCG vaccination,[17] and 3–7 weeks following known *M. tuberculosis* exposure.[18] Furthermore, in The Gambia, TB cases that commence treatment tend not to be removed from their family compounds and remain infectious for a period of time after starting their medication. For these two reasons, a repeat screening procedure is indicated in Gambian TB case contacts that are initially negative. Here we present evidence that tuberculin skin test conversion after 3 months in such individuals is relatively increased in certain subgroups, consistent with the presence of boosting. Second, we have shown that ELISPOT is not subject to boosting by the tuberculin skin test in community volunteers. Third we demonstrate that the ELISPOT behaves in the way one would expect if it were able to distinguish those with true conversion from those with boosting.

While tuberculin test conversion in this study was associated with closer proximity to a known TB case, suggesting some genuine conversion, increased conversion with increasing age and in those with a BCG scar, provide indirect and direct evidence respectively of boosting of immunity to previous, largely non-tuberculous, mycobacterial exposure. Indeed, others have shown that individuals positive on an initial tuberculin test are much more likely to progress to TB disease than those positive only on a second test.[19], [20]

We found that individuals who were tuberculin skin test negative and ELISPOT positive at screening were more likely to undergo tuberculin test conversion. Whether this reflects a shorter incubation period for the ELISPOT test, or something else, requires further study. It will also be important to determine whether ELISPOT converters are more or less likely to be initially tuberculin test positive at recruitment and if ELISPOT conversion occurs in the absence of skin test conversion. That 9 (36%) of 25 tuberculin converters who were initially ELISPOT positive had ELISPOT reversion is of concern. ELISPOT reversion is not unexpected in theory: the ELISPOT assay detects recently activated lymphocytes with immediate effector function and effector-memory cells that persist for a limited time in circulation once antigen is cleared.[21], [22] If *M. tuberculosis* is either cleared from the body or transits into dormancy, where ‘early secreted’ antigens may not be consistently secreted, one would expect ELISPOT test reversion to occur. However, it is likely that some individuals simply do not mount a strong T cell response to ESAT-6 or CFP-10 and the T cell response may vary with evolution of an infection.[23] There is an urgent need for studies of repeated ELISPOT tests in a large number of TB case contacts. Therefore we have established a cohort of consecutively recruited TB case contacts in The Gambia that have a repeat ELISPOT test after 3 and 18 months, and a repeat skin test after 18 months.

Our ELISPOT results suggest that approximately one third of tuberculin conversion in Gambian TB case contacts is due boosting of immunity to non-tuberculous mycobacterial exposure. It is important that a test which can identify individuals who have boosting can also identify as many as possible of those who undergo true conversion. Both secondary cases with repeat ELISPOT results were strongly ELISPOT positive at diagnosis, and the ELISPOT test probably has sensitivity of 70–85% for the diagnosis of *M. tuberculosis* infection from recent exposure in The Gambia,[12] and 80–90% for the diagnosis of tuberculosis disease.[16] Sensitivity to disease may vary between certain groups. For example, the commercial ‘T-spot’ ELISPOT assay detected only 9 (69%) of 13 cases with pulmonary TB but all 11 cases of extra-pulmonary TB in a recent study from Italy.[8]

The definition of tuberculin test conversion has been the subject of considerable debate. We chose a cut-off used previously in The Gambia,[15], [24] in addition to a criterion of at least a 6 mm increase in induration between tests. This criterion is recommended on the basis that chance variation in the tuberculin test reading results in less than 6 mm of induration in over 95% of individuals.[25], [26] Using an alternative 10 mm increase in induration, only 3 fewer contacts would have been included as skin test converters and the findings of the study were not significantly altered (data not shown). In The Gambia we have applied mathematical tools to ELISPOT results in TB cases and their household contacts to identify a cut-off of 8 spots/well (40 spots/million cells) above the negative control well when using two antigens.[12] While we cannot be sure that we have identified exactly the right criterion for ELISPOT conversion, only 2 more individuals would have been categorised as ELISPOT converters if no criterion for an increase in spot count had been set, other than for the test to turn positive; and only 2 individuals would have been re-categorised as non-converters if a 10 spot increase had been specified.

At the present time, treatment of *M. tuberculosis* infection is prohibitively expensive and impractical in many developing countries. In the United States, it is advised that those case contacts who are initially screened during the ‘window period’, and have a negative result, should have a repeat tuberculin test - all those that become tuberculin positive are offered prophylactic treatment.[27] Any possible way to help distinguish TB case contacts that would benefit from prophylactic treatment and those that wouldn't is therefore of relevance in both developing and developed country settings. Further longitudinal studies are indicated to help confirm that our findings are of practical importance in this regard, and to address the relevance of ELISPOT reversion in particular. It does appear that an ELISPOT assay may have a niche in exposing boosting of immunity to non-tuberculous mycobacterial exposure.
